# Supplementary material for: Safety Evaluation of Heavy Metal Contamination and Pesticide Residues in Coix Seeds in Guizhou Province, China
Source: Foods. 2022 Jul 31;11(15):2286. doi: 10.3390/foods11152286 (PMC9367953; doi:10.3390/foods11152286)
Supplement: Supplementary file 1 [file foods-11-02286-s001.zip › foods-1806888-supplementary.pdf]

Supplementary Materials: Safety Evaluation of Heavy Metal Contamination and Pesticide Residues in Coix Seeds in Guizhou Province, China

Jiaxing Yu, Xiangui Wang, Xiaolong Yao, Xiaomao Wu

Table S1. The scoring standard for the risk ranking index of pesticide residues in coix seed.

Index	Value	Score
Toxicity	Low	2
	Moderate	3
	High	4
	Severe, carcinogenic	5
Toxic effect	＞1×10-2	0
	1×10-4 ~ 1×10-2	1
	1×10-6 ~ 1×10-4	2
	<1×10-6	3
Dietary proportion	＜2.5	0
	2.5 ~ 20	1
	20 ~ 50	2
	50 ~ 100	3
Usage frequency	＜2.5	0
	2.5 ~ 20	1
	20 ~ 50	2
	50 ~ 100	3
High exposure population	None	0
	Not too possible	1
	Probably	2
	With or without relevant data	3
Residual level	Not detected	1
	＜1MRL	2
	≥1MRL	3
	≥10MRL	4
